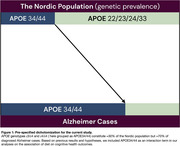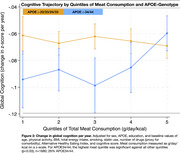# APOE‐Genotype Modifies the Association of Meat Consumption with Cognitive Decline: Longitudinal Analyses of The Swedish National Study on Aging and Care, SNAC‐K

**DOI:** 10.1002/alz70860_107412

**Published:** 2025-12-23

**Authors:** Jakob Norgren, Adrián Carballo‐Casla, Anne Börjesson‐Hanson, Maria Eriksdotter, Erika J Laukka, Sara Garcia‐Ptacek

**Affiliations:** ^1^ Karolinska Institutet, Stockholm, Sweden; ^2^ Aging Research Center, Karolinska Institutet and Stockholm University, Stockholm, Sweden; ^3^ CIBER of Epidemiology and Public Health (CIBERESP), Madrid, Spain; ^4^ Karolinska University Hospital, Stockholm, Sweden; ^5^ Stockholm Gerontology Research Center, Stockholm, Sweden

## Abstract

**Background:**

The gene variant 𝜀4 of *APOE* (APOE4) increases Alzheimer risk and may provide adaptation to animal‐ versus plant‐based food. Our aim was to test the hypothesis that higher meat consumption has a more favorable impact on cognitive health in subjects with APOE34/44 compared to APOE22/23/24/33 (Figure 1).

**Method:**

We included data from a longitudinal population‐based study comprising 1680 dementia‐free subjects aged ≥60 years, with information on APOE‐genotype, habitual diet, and longitudinal cognitive measurements (age 70±9 years, 63% females, 26% APOE34/44). Diet was measured with validated food frequency questionnaires at years 0, 3, and 6, and mean values were used in the analyses. The outcome was a linear trajectory of global cognition (mean z‐score from 4 subdomains) over 15 years (6‐year retention: 90%; 12‐year: 59%). Linear regression was used to estimate the association of meat consumption (in g/day/kcal) with said cognitive trajectory, adjusted for potential confounders. Interaction terms with APOE34/44 were included.

**Results:**

Higher total meat consumption (red meat: 51%, poultry: 20%, processed meat: 29%), was associated with a better cognitive trajectory only in the APOE34/44 stratum (β per 1 z‐unit increase: 0.011; CI: 0.004 to 0.018 versus –0.003; CI: –0.008 to 0.003; P for interaction = 0.002). The association was primarily driven by episodic memory (β =0.020; CI:0.007 to 0.033). In all other sub‐domains (i.e., semantic memory, verbal fluency, perceptual speed), associations were weaker but in the same direction. Associations with global cognition were consistent when comparing the highest (median: 783 g/week) versus lowest (240 g/week) quintile of total meat consumption (β=0.034; CI: 0.009 to 0.060) (Figure 2). The strongest association in the highest quintile was also with the episodic memory domain (β=0.053; CI: 0.012 to 0.097).

**Conclusion:**

Among older *APOE* 𝜀34 and 𝜀44 genotypes, lower meat consumption was associated with faster cognitive decline, while higher meat consumption resulted in similar cognitive change trajectories as those of *APOE* 𝜀22/23/24/33 genotypes. Our results support the hypothesis that the ancestral gene variant—APOE4—provides adaptation to meat consumption, at least from the perspective of cognitive health. Further studies on broader health outcomes and dementia conversion are warranted.